# A Constitutive Model for Alginate-Based Double Network Hydrogels Cross-Linked by Mono-, Di-, and Trivalent Cations

**DOI:** 10.3390/gels7010003

**Published:** 2020-12-30

**Authors:** İsmail Doğan Külcü

**Affiliations:** Department of Materials Science and Engineering, İzmir Katip Çelebi University, 35620 İzmir, Turkey; ismaildogan.kulcu@ikc.edu.tr

**Keywords:** polysaccharide hydrogels, inelastic features, polymer chain behavior, constitutive modeling

## Abstract

In this contribution, a micro-mechanically based constitutive model is proposed to describe the nonlinear inelastic rubber-like features of alginate-based double network hydrogel cross-linked via various counterions. To this end, the lengthening of the polysaccharide polymer chain after a fully stretched state is characterized. A polymer chain is firstly considered behaving entropically up to the fully stretched state. Then, enthalpic behavior is accounted for concerning the following lengthening. To calculate enthalpic behavior, the macroscopic material properties, such as elastic modulus, are integrated into the proposed model. Thus, a new energy concept for a polymer chain is proposed. The model is constituted by the proposed energy concept, the network decomposition model, the Arruda–Boyce eight chain model and the network alteration theory. The model is compared against the cyclic tensile test data of alginate-based double network hydrogels cross-linked via mono-, di-, and trivalent cations. Good agreement between the model and experiments is obtained.

## 1. Introduction

In the field of bioengineering, biocompatible materials with particular and controllable structures are frequently required [1]. Such materials are utilized for applications, such as drug delivery [2], bulking agent [3] or extracellular matrix [4]. In this regard, hydrogels are one of the most important candidates in bioengineering.

Hydrogels are a class of three-dimensional polymer networks consisting of hydrophilic polymer chains and containing water up to 99.9% of their dry weights [5]. Due to their biocompatibility, water absorption capacity and environment-sensitive structures, they have attracted attention since the beginning of the 1950s in various fields ranging from cosmetics to the food industry and electronics to bioengineering applications [6]. Particularly, polysaccharide hydrogels are used as biomaterials in various applications due to their mimic ability to extracellular matrix role of living tissues [7]. However, the mechanical weakness of conventional hydrogels has obstructed the usage of these materials in many implementations requiring a load-bearing material [8].

Since the beginning of the last decade, many attempts have been carried out to strengthen and toughen hydrogels [9]. Nano-composite gels [10], slide ring gels [11], double network hydrogels (DNH) [12] are some of which proposed in the literature and show very promising mechanical properties for load-bearing applications.

Among tough hydrogels, a double network concept, which has so far provided one of the best mechanical properties for hydrogels, was proposed by Gong et al. [12]. A DNH is made up mainly of highly cross-linked polyelectrolyte and sparsely cross-linked neutral polymer networks. The polyelectrolyte network acts as a skeleton of the polymer matrix, whereas the neutral network keeps the structure together. To achieve tough hydrogels within this concept, some additional conditions with regard to cross-linked networks, cross-linking density and molar ratio of the networks should be satisfied [13]. However, these hydrogels also demonstrate inelastic features such as stress softening, necking, yielding and do not show self-healing after deformation, which is very critical for biological applications [14,15].

Polysaccharides are abundant materials in nature. Cellulose, which is the most predominant material found in nature, is also a class of polymeric polysaccharides. Most of the polysaccharides can be in the form of hydrogel due to their natural properties [16]. Alginate, agarose, chitosan, hyaluronic acid are some of the polysaccharides forming hydrogel. Due to their biological adaptation to the body as well as structural similarities to living tissues, polysaccharide hydrogels are important for biological implementations. In literature, numerous studies have been carried out about polysaccharide hydrogels for different fields [7,17,18,19].

To overcome the mechanical weakness of polysaccharide hydrogels, toughening methods, some of which are mentioned above, are used [20]. Sun et al. [21] synthesized an alginate/PAAm DNH showing time and temperature-dependent self-healing after deformation. One of the most important inelastic features of natural tough hydrogels mimicking living tissues is self-healing. In this respect, the cross-linking procedure of tough hydrogels is critical. Generally, chemical procedures for self-healing tough hydrogels were based on changing the cross-linkages in the highly cross-linked polyelectrolyte network from covalent bonds that break in an irreversible way to weaker interactions such as ionic metal complexes which break in a reversible way. For example, the polyelectrolyte alginate network is formed by complex formation between calcium ions and the carboxylic acids of the alginate [21]. This alginate network was used in alginate/PAAm DNH with time and temperature-dependent self-healing behavior. Later, alginate/PAAm DNH is improved by using ion-sensitive segments to control the dimension and shape fidelity of the material [22]. To achieve better material response under deformation, different divalent and trivalent cations are utilized instead of Ca^+2^ as well [23,24,25]. The result of these studies shows that cations having a larger ion radius, such as Fe^+3^, provide better stability in the biological environment and better mechanical features of the material.

Under large deformation DNH generally exhibits J- and S-shaped nonlinear stress behavior along with inelastic features similar to the rubber-like materials. To characterize inelastic features, such as the Mullins effect and hysteresis, background information for rubber-like materials is a useful tool. Constitutive models within the perspective of rubber elasticity are widely used to model complicated behaviors of rubber-like materials [26,27,28,29,30]. Rubber-like behaviors of DNH are also described using different concepts of the rubber elasticity. First models assumed the damage in DNH as a chain breakage [14,31]. Later, Zhao [32] decomposed the polymer matrix into short and long polymer chains and used Arruda–Boyce eight chain model [33] and the network alteration theory [34] to characterize the damage in DN hydrogel and the stress softening. Külcü [35] proposed a model for the stress softening and self-healing behavior of alginate-based DNH by using the network decomposition concept [29] and thermal effects in DNH put into a thermal chamber for self-healing. Recently, Morovati and Dargazany represented a micro-mechanical constitutive model for the stress softening in DNH. Their model is based on the network evolution concept proposed by Dargazany and Itskov [27] and shows good agreement with experimental data. Mao et al. [36] constituted a viscoelasticity model for the complicated behavior of DNH under large deformation. Their model successfully describes the viscoelastic features as well as the stress softening in DN hydrogels.

In this contribution, a micro-mechanically based model is proposed to describe the enthalpic behavior of a polysaccharide polymer chain as well as an entropic one and the effect of various types of ionic cross-linkages in polysaccharide-based DNH. To constitute the present model, a new form of an energy function of a single polymer chain is introduced. Also, the Arruda–Boyce eight chain model [33], the network decomposition approach [27,29] and the network alteration concept [37] are pursued in the model.

## 2. Results and Discussion

The proposed model consists of eight material parameters for inelastic and hyperelastic polymer networks. Six material parameters belong to the polymer network exhibiting inelasticity, of which five of them are utilized for the entropic behavior while one parameter is taken for the enthalpic behavior. Two material parameters are accounted for the hyperelastic part of the model. Table 1 shows the material parameters along with their description. In the verification of the model with experimental data, the Levenberg-Marquardt algorithm has been used to find the values of the material parameters.

**Figure 1:**
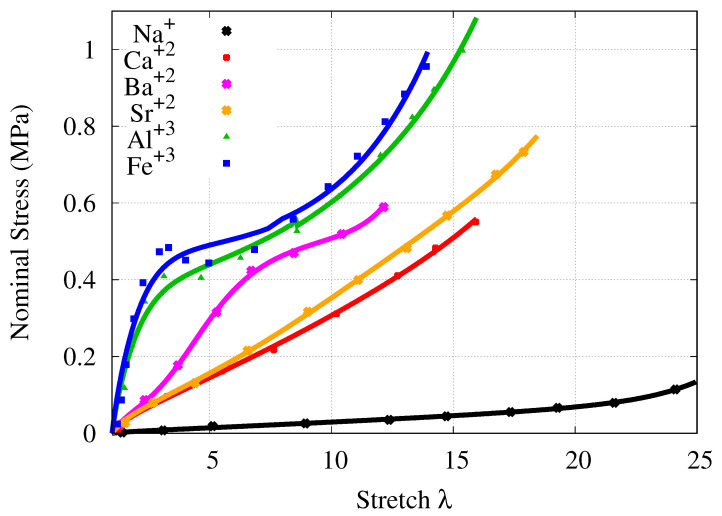
Comparison of the model with the stress-stretch curves of various alginate-PAAm double network hydrogels (DNHs), which are elongated to rupture [23]. (Lines: proposed model. Points: experimental data.)

Figure 1 illustrates the comparison of the model behavior against the stress-stretch curves of various alginate-PAAm DNHs elongated to rupture. All comparisons agree well with the experimental data of alginate-PAAm DNH cross-linked via different types of cations. The model is able to describe J- and S-shaped behavior of alginate-based DNHs.

**Figure 2:**
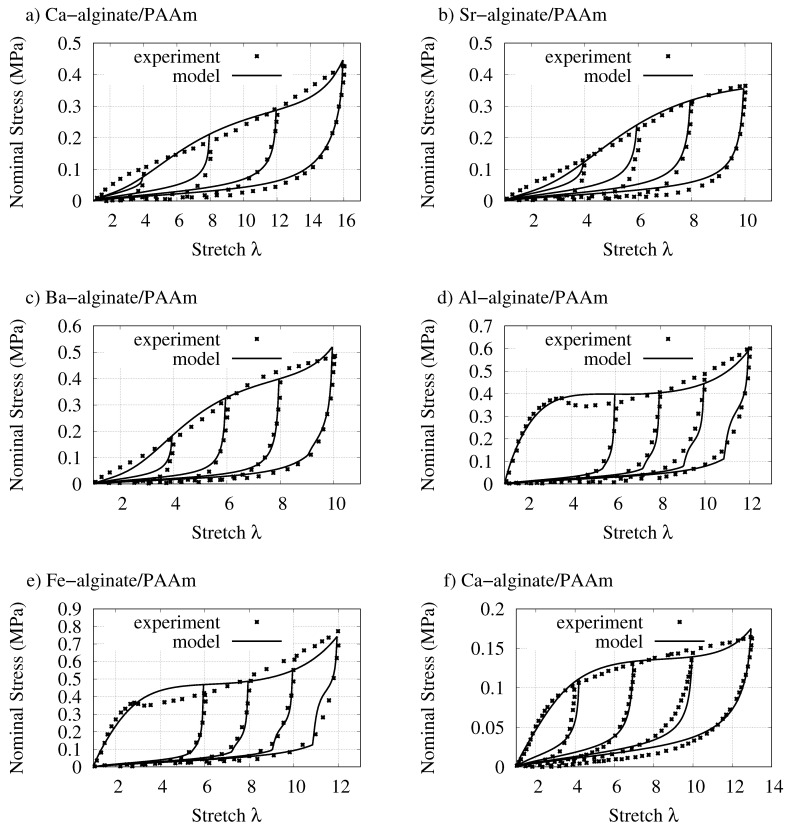
Comparison of the model with the quasi-static cyclic tensile test data of various alginate-PAAm DNHs. (**a**–**e**): by Yang et al. [23] and (**f**): by Sun et al. [21].

Figure 2 demonstrates the validation of the model with experimental data of alginate-PAAm DNHs subjected to the quasi-static cyclic tensile deformation. For different types of DNHs the proposed model achieves quantitatively and qualitatively good agreement with experimental data. As the alginate-PAAm DNH cross-linked via Na^+^ does not exhibit substantial stress softening according to the experimental data, the cyclic tensile deformation behavior of this material is not shown.

**Figure 3:**
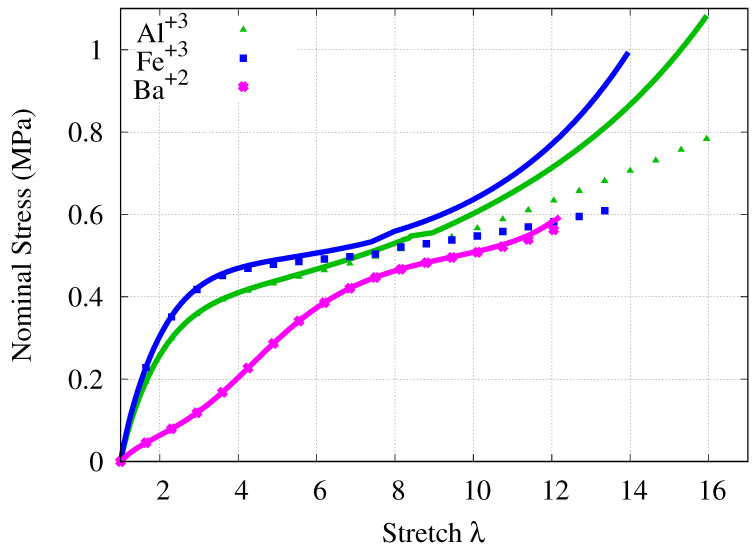
The effect of the enthapic part. (Lines: proposed model. Points: proposed model without enthalpic part.)

Lastly, to depict the effect of the new contribution, the enthalpic part is removed from the model. Then, the sum of the stress contributions of the entropic and hyperelastic parts are compared with the proposed model (see Figure 3). The modeling results for Fe${}^{+3}$, Al${}^{+3}$ and Ba${}^{+2}$ are utilized, as algiate/PAAm DNHs cross-linked via these counterions exhibit relatively more complicated behavior. The influence of the enthalpic part is clearly seen in hardening part of the material showing S-shaped behavior.

It should be considered here that materials are subjected to tensile deformation, which is more than ten times of initial length. Although such huge deformation may result in a dramatic change in the material behavior and material structure, the proposed model is able to achieve a considerable match with the experimental data. The error margin of the model against experimental data is rather low. However, a yielding-like behavior is shown in the experimental data of DNHs cross-linked via trivalent cations during the deformation (see [23] and Figure 2d,e). To physically describe this behavior in the modeling, approaches of elastoplasticity might be useful to have a better characterization of this types of DNH.

## 3. Conclusions

In this study, a micromechanical constitutive model is proposed for polysaccharide-based DNH cross-linked via mono-, di- and trivalent cations. Due to the lengthening of the polysaccharide chain after a fully-stretched state, the free energy of a single chain is considered to involve enthalpic behavior as well as entropic one in the model. As a modeling tool, the Arruda–Boyce eight chain model [33], the network decomposition concept [38], the network alteration theory [37] and Hooke’s law are used. First, the DNH matrix is decomposed into polyelectrolyte and neutral networks, where the latter one is assumed to be a purely elastic material. Then, the polyelectrolyte network is decomposed into entropic and enthalpic parts. For entropic chain behavior, the Langevin chain is used. To consider enthalpic behavior after the fully-stretched state, a straight polymer chain is assumed to be a cantilever and relatedly Hooke’s law is utilized to describe stress contribution of the polymer chain due to its lengthening. Reorganization of the DNH matrix is taken into account by the network alteration theory. Finally, the model is compared against various DNH types. Good agreement between the model and experimental data is obtained.

## 4. Constitutive Modeling

A DNH consists of polyelectrolyte and neutral polymer networks [12]. While the latter is made up of loosely cross-linked long polymer chains, highly cross-linked polymer chains form the polyelectrolyte network. Alginate-PAAm hydrogel (APH) considered in the present manuscript is synthesized using the DNH concept [21,23]. Alginate of APH is a class of polysaccharides and acts as a polyelectrolyte network cross-linked via various types of cations in the DNH matrix. Polyacrylamide of APH is the neutral network and mainly ensures the stability of the polymer matrix. Within the perspective of the constitutive modeling, the strain energy (Ψ) of a DNH can be investigated by decomposing the DNH matrix into polyelectrolyte and neutral polymer networks as

(1) $$\Psi^{M}=\Psi^{P}+\Psi^{N},$$

where superscripts ${•}^{M}$, ${•}^{P}$ and ${•}^{N}$ denote the DNH matrix, polyelectrolyte and neutral polymer networks, respectively.

**Figure 4:**
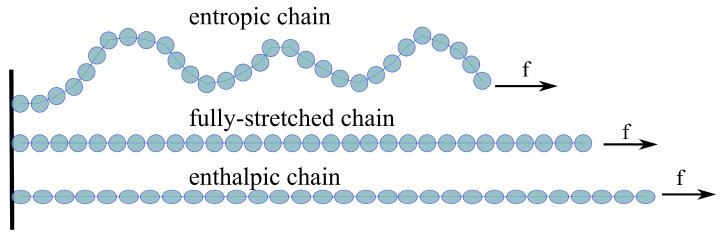
Illustration of the deformation of a polysaccharide chain.

In studies dealing with constitutive modeling, the energy of a polymer chain in micro-scale is mainly dominated by entropy [26,30]. The Langevin approximation is one of the most common approaches to model the entropic energy of a polymer chain [27,33]. However, in case of polysaccharides, during the deformation each monomer experiences a lengthening by about 10% [39] (see Figure 4). This fact necessitates a modification in the treatment of the modeling of a polymer chain, which is to consider the enthalpic behavior of a polymer chain in addition to the entropic one. A modified freely jointed chain (m-FJC) concept has been proposed to involve enthalpic and entropic energies simultaneously [40,41]. However, the derivation of m-FJC is mathematically complex and requires an approximation. This fact suspends the modeling approach from the physical motivation in the micro-scale. In the current paper, a micro-mechanically motivated model considering enthalpic and as well as entropic behaviors of a polymer chain is proposed. To this end, $\Psi^{P}$ is decomposed into entropic (${•}^{S}$) and enthalpic (${•}^{H}$) and $\Psi^{M}$ is rewritten as

(2) $$\Psi^{M}=\Psi^{S}+\Psi^{H}+\Psi^{N}.$$

Assuming the polyacrylamide network is purely elastic [15], a hyperelastic model can be used to produce its mechanical behavior. $\Psi^{N}$ is thus given by [42]

(3) $$\Psi^{N}=\mu \left[f\left(I_{1},\alpha \right)+f\left(I_{2},-\frac{\alpha }{16}\right)+\ln\left(\frac{1}{\alpha }f\left(I_{1},1\right)+1\right)\right],$$

where $I_{1}$ and $I_{2}$ are the first and second invariants of the right Cauchy-Green deformation tensor, respectively, μ is the shear modulus, α is the scalar material parameter and

$$f\left(x,y\right)=\frac{\alpha }{y}\left[e^{y[x-3]}-1\right].$$

Introducing the number of polymer chains in the alginate network $N^{P}$, $\Psi^{P}$ is expressed as

(4) $$\Psi^{P}=N^{P}\psi^{P}=N^{P}\left(\psi^{S}+\psi^{H}\right).$$

To model the entropic behavior of the alginate network, the Arruda–Boyce eight chain model [33] is used due to its relatively simple and efficient mathematical expression. In the Arruda–Boyce model, a unit cube from a polymer network has eight chains along the half diagonals of the cube. Deformation of the unit cubes results in the stretching of the polymer chains in a network by the same ratio.

The entropic energy of a single chain is captured by the Langevin statistics in the Arruda–Boyce eight chain model and can be expressed as

(5) $$\psi^{S}=kTn\left(\frac{\bar{r}}{n}\beta +\ln\frac{\beta }{\sinh\beta }\right),$$

where *k* is the Boltzmann constant, *T* is the absolute temperature, *n* is the segment number, $\bar{r}$ is the normalized average contour length and β is the inverse Langevin function. To approximate β, the Puso approximation [43] is used.

**Figure 5:**
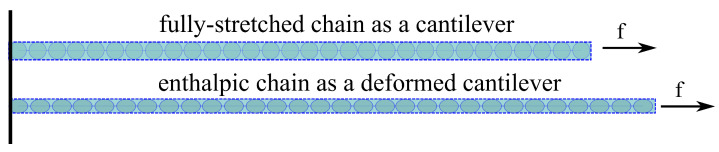
Deformation of a polysaccharide chain after fully-stretched state.

**Figure 6:**
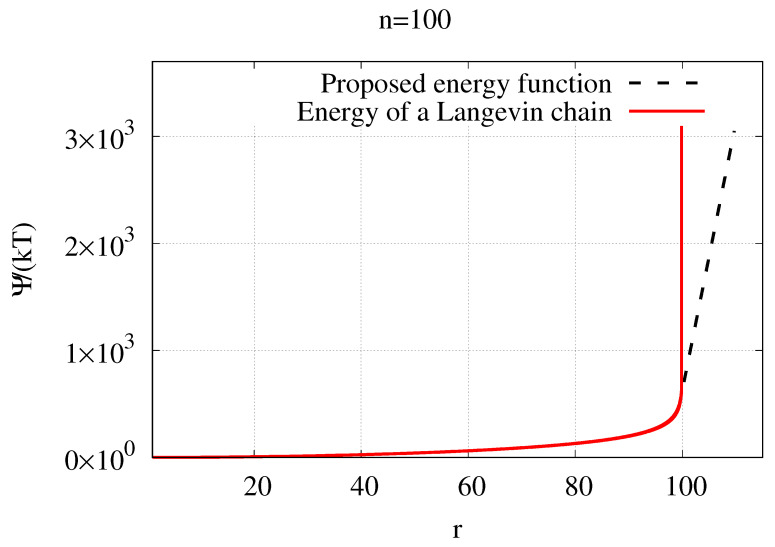
Comparison of $\psi^{P}$ against the energy of a Langevin chain. (Note that $k^{-1}T^{-1}\approx N^{P}$ is assumed.)

As the stretch ratio is assumed to be the same for all chains, a fully stretched polymer chain can be considered as a cantilever to describe the enthalpic behavior (see Figure 5). Therefore, Hooke’s law can be integrated into the enthalpic energy of a single chain as

(6) $$\psi^{H}=\frac{E}{N^{P}}\epsilon ,$$

where *E* is the elastic modulus of the polymer matrix and taken from the experimental data [23], ϵ is the strain, which a polymer chain undergoes. Figure 6 shows the comparison between the entropic energy of a Langevin chain and the proposed energy function for $\psi^{P}$.

**Figure 7:**
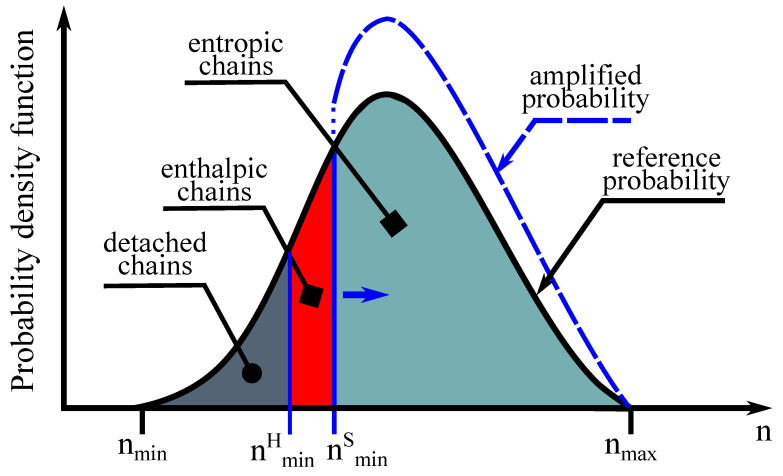
Schematic view of the probability density function over the set of available chain in a particular direction (D) during deformation.

A probability density function is accounted for a network exhibiting inelastic behavior to define the network evolution under deformation (see Figure 7). To this end, a set of available chain D is firstly specified as

(7) $$D=\{n|n_{min}\lambda \leq n\leq n_{max}\},$$

where $n_{min}$ and $n_{max}$ denote minimum and maximum segment numbers in a network, respectively. $n_{max}$ is taken as a material parameter, where is $n_{min}$ is given by

(8) $$n_{min}^{S}=n_{min}\lambda =\nu \bar{r}\lambda ,$$

where ν is a material parameter utilized to prevent singularity at the fully stretched state of a Langevin chain. Also, note that the affine deformation is taken into account. Thus, macro-stretch Λ is equal to λ.

D indicates the chain stretch and entropic behavior. However, fully stretched chain is deformed further due to deformation of polymer segments. Thus, a new set of available chain for entropic behavior $D^{H}$ is written as

(9) $$D^{H}=\left\{n|n_{min}^{H}\lambda \leq n\leq n_{min}^{S}\right\},$$

where $n_{min}^{H}$ is the minimum segment number of a polymer chains showing enthalpic behavior. Considering 10% strain value for a fully-stretched polymer chain [39], $n_{min}^{H}$ is represented as

(10) $$n_{min}^{H}=\frac{10}{11}n_{min}^{S}.$$

The Gaussian distribution gives a sufficient approximation if a polymer network is polymerized randomly. Assuming the damage in a network under loading takes place with respect to the chain length from the shortest chain to longer ones [29] and considering cross-linking density in the probability distribution [27,28,35], the probability density function (PDF) is written as,

(11) $$P\left(n,\bar{r},\kappa \right)=\kappa \sqrt{\frac{6}{\pi n}}e^{G_{1}},$$

where κ is the material parameter describing the cross-linking density of a polymer network and

$$\begin{array}{ccc} & & G_{1}=-G_{2}-\kappa \sqrt{\frac{24}{\pi }}\left[G_{3}+\sqrt{n}e^{-G_{2}}-e^{-G_{2}n}\right],\\ & & G_{2}=\frac{3{\bar{r}}^{2}}{2n},\\ & & G_{3}=\sqrt{G_{2}n\pi }\left[\mathrm{erf}\left(\sqrt{G_{2}}\right)-\mathrm{erf}\left(\sqrt{G_{2}n}\right)\right]. \end{array}$$

To normalize the PDF, the network alteration theory, which is to assume constant active segment number in a network, [37] is taken into account as

(12) $$\Phi =\frac{1}{\int_{D}P\left(n,\bar{r},\kappa \right)ndn}.$$

The total energy of the alginate network is represented by

(13) $$\Psi^{P}=\Phi \left(N^{P}\int_{D}P\left(n,\bar{r},\kappa \right)\psi^{S}dn+E\int_{D^{H}}P\left(n,\bar{r},\kappa \right)dn\right).$$

Macroscopic energy of a polysaccharide-based DNH is thus written as

(14) $$\begin{array}{ccc} \Psi^{M} & & =\Phi \left((N^{P}\int_{D}P\left(n,\bar{r},\kappa \right)\psi^{S}dn+E\int_{D^{H}}P\left(n,\bar{r},\kappa \right)dn\right)\\ & & +\mu \left[f\left(I_{1},\alpha \right)+f\left(I_{2},-\frac{\alpha }{16}\right)+\ln\left(\frac{1}{\alpha }f\left(I_{1},1\right)+1\right)\right]. \end{array}$$

Finally, the first Piola-Kirchhoff stress tensor P of the DNH matrix based on the incompressibility condition is given by

(15) $$\begin{array}{cc} P & =\frac{\partial \Psi_{M}}{\partial F}=\frac{\partial \Psi^{P}}{\partial F}+\frac{\partial \Psi^{N}}{\partial F}-pF^{-T}, \end{array}$$

where F is the deformation gradient.

## Figures and Tables

**Table 1:** List of material parameters and their description.

Material Parameter	Description
*Inelastic (entropic)*	
$n_{max}$	maximum segment number in the network
$\bar{r}$	normalized average contour length of the polymer chain
κ	cross-linking density of the network
$N^{P}$	number of active polymer chains in the polyelectrolyte network
ν	sliding ratio of a chain during rupture
*Inelastic (enthalpic)*	
*E*	elastic modulus of the material
*Hyperelastic*	
μ	shear modulus of the polymer network
α	scalar parameter

## Data Availability

The data that support the findings of this study are available from the corresponding author, upon reasonable request.
